# The association between recreational physical activity and depression in the short sleep population: a cross-sectional study

**DOI:** 10.3389/fnins.2023.1016619

**Published:** 2023-05-25

**Authors:** Yanwei You, Mengxian Wei, Yuquan Chen, Yingyao Fu, Alimjan Ablitip, Jianxiu Liu, Xindong Ma

**Affiliations:** ^1^Division of Sports Science and Physical Education, Tsinghua University, Beijing, China; ^2^School of Social Sciences, Tsinghua University, Beijing, China; ^3^Institute of Medical Information/Medical Library, Chinese Academy of Medical Sciences & Peking Union Medical College, Beijing, China; ^4^Beijing Jianhua Experimental Etown School, Beijing, China; ^5^Vanke School of Public Health, Tsinghua University, Beijing, China; ^6^IDG/McGovern Institute for Brain Research, Tsinghua University, Beijing, China

**Keywords:** recreational physical activity, Patient Health Questionnaire-9, short sleep, dose-response, epidemiology

## Abstract

**Background:**

Short sleep is more common in the modern society. Recreational physical activity (RPA) like exercise brings both mental and physiological benefits to depression; paradoxically, lack of sleep is harmful. Evidence on the association between RPA and depression in the short sleep population is limited.

**Methods:**

Participants with short sleep condition from the National health and Nutrition Examination Surveys (NHANES 2007–2018) were included in the present study. Short sleep condition was defined as ≤ 7 h per night. Sleep duration and RPA status were self-reported in NHANES by the Physical Activity Questionnaire using a 7-day recall method. Multivariable logistic regression was applied to evaluate the association between RPA and depression. Additionally, the non-linear relationship between RPA and depression was evaluated using the threshold effect analysis and restricted cubic spline.

**Results:**

This cross-sectional study comprised 6,846 adults' data, and the weighted participants were 52,501,159. The weighted prevalence of depression was higher in females, which took up 65.85% of all depression patients. In fully adjusted models, sufficient volume of RPA was associated with lower depression risks, with OR (95% CI) =0.678 (0.520, 0.883). Further analysis revealed a U-shaped association between RPA and incident depression, and the inflection point was 640 MET-minutes/week. When RPA <640 MET-minutes/week, increased RPA was associated with lower risk of incident depression, with OR (95% CI) = 0.891 (0.834, 0.953). When RPA ≥ 640 MET-minutes/week, the benefits of RPA seemed to be not significant, with OR (95% CI) = 0.999 (0.990, 1.009).

**Conclusion:**

Our findings observed associations between RPA condition and incident depression in the short sleep population. Moderate RPA was beneficial to maintain mental health and associated with lower incidence of depression for short sleepers, but excessive RPA might increase the risk of depression. For general short sleepers, keeping the RPA volume approximately 640 MET-minutes/week was beneficial to lower risks of depression. Gender difference should be considered as an important factor for further studies to examine these relationships and explore mechanisms.

## 1. Introduction

Depression is a disabling psychiatric condition and is now becoming one of the leading causes of global health burden (Moussavi et al., 2007; Malhi and Mann, 2018). There are over 300 million people, or 4.4% of the global population, suffering from depression (Ferenchick et al., 2019). As a chronic mental disorder that can affect both physical and psychological health, depression holds people back from their full potential and is associated with premature mortality from suicide and other disease, including cardiovascular disease (Carney and Freedland, 2003), diabetes (Zhang et al., 2005), and hypertension (Kuo and Pu, 2011). In addition, gender seems to be an important factor that affects the prevalence of depression, and depression in women is about twice as high as men (Kuehner, 2003). However, little is known about the positive factor in the prevention of depression, and current evidence tends to prove that lifestyle factors including sleep and physical activity are associated with depressive symptoms.

Short sleep condition is more common in sync with the accelerating pace of work and life in the modern society. As a basic part of a person's daily routine, sleep is fundamental to individual's whole life cycle health (Buysse, 2014; Grandner, 2020). The American National Sleep Foundation has recommended a 7- to 9-h sleep duration for adults (Chaput et al., 2018), and referring to this criteria, sleep duration less than 7 h is regarded as short sleep status. Nevertheless, there were scarcely 50% of adults in the US reported a habitual sleep time falling within the recommended sleeping hour (Covassin and Singh, 2016), and this trend was also found in other developed countries (Bin et al., 2012).

As an emerging strategy to prevent and counter depression, physical activity did not get enough attention in the early years but has received more and more attention recently. Physical exercise has been acknowledged globally for its multifaceted health benefits, both from physiological and psychological dimensions, including improved muscle quality (You et al., 2023a), a longer lifespan (Gremeaux et al., 2012), and enhanced cognitive function (You et al., 2023b). Generally, several meta-analyses (Kvam et al., 2016; Korczak et al., 2017) suggested that recreational physical activities (RPAs), such as sports and exercise, were useful ways for improving depressive symptoms and preventing other mental disorders. However, there was also evidence that the antidepressant effects of exercise were not significant (Krogh et al., 2017). The results of meta-analysis should be caution due to the underlying differences about study designs, definition of depression, intervention types of exercise, or other important variables that could not be considered.

Collectively, preliminary findings supported the widely held belief that being physically active was beneficial to counter depression, while not getting enough sleep was harmful. However, it remained doubtful that what is the relationship between recreational physical activity and depression in the short sleep groups. Some of this controversy was partly due to the lack of dose–response exploration in this topic and what an appropriate amount of physical activity should be. Hence, it was of great importance to examine the risk–benefit relationship between health benefits of RPA and the potential risks of short sleep to depression.

To our knowledge, there was no existing research that explicitly explored the impact of recreational physical activity (RPA) on depression symptoms in the short sleep population. In this study, by using a general sample from the National Health and Nutrition Examination Survey (NHANES), we sought to (Moussavi et al., 2007) examine the relationship between RPA and depression symptoms in the short sleep group and (Malhi and Mann, 2018) quantify the form of this association and further assess the relationship by gender stratification.

## 2. Methods

### 2.1. Study participants

The National Health and Nutrition Examination Survey (NHANES), a representative survey of the national population in the United States, was conducted by the Centers for Disease Control and Prevention (CDC). By applying a complicated, multistage, and probabilistic sampling method, this survey can provide a wealth of information about the nutrition and health of the overall US population (Curtin et al., 2013; Patel et al., 2016). The National Center for Health Statistics ethics review board approved the NHANES protocols with the written informed consent of all individuals participating in.

This cross-sectional study analyzed the data from 2007 to 2018, representing six cycles of the NHANES. A total of 34,770 adult participants over the age of 20 were included in the analysis after excluding participants under the age of 20 (*n* = 24,106). After that, individuals with sleep duration over 7 h (*n* = 16,054) were excluded, leaving 18,716 for further analysis. Subsequently, participants with missing physical activity data (*n* = 7,854) and with missing depression data (*n* = 4,016) were excluded. Finally, 6,846 participants were analyzed in this research (Supplementary Figure S1).

### 2.2. Measurement

#### 2.2.1. Outcome ascertainment

Depression, the outcome in this research, was assessed with the Patient Health Questionnaire-9 (PHQ-9). The PHQ-9 was one of the most widely used depression screening tools to assess depressive symptoms. It consisted of nine items matching the Diagnostic and Statistical Manual of Mental Disorders, Fourth Edition (DSM-IV) criteria. This short screening questionnaire scored the signs and symptoms of depression in nine questions on a scale from “0” (not at all) to “3” (nearly every day) (Kroenke et al., 2001; Liu et al., 2017). PHQ-9 in the NHANES was tested during the face-to-face MEC interview to evaluate depression conditions over the past 2 weeks. Item scores for each participant were summed to form a total score (range: 0–27). It was recommended that PHQ-9 score >10 can be used as a screening cut-point for depression, with 88% sensitivity and specificity to diagnose the major depression (Kroenke et al., 2001). Therefore, we defined the PHQ-9 scores <10 as no depression and ≥ 10 as depression.

#### 2.2.2. Exposure measurement

Sleep duration on usual weekday or workday was self-reported by participants. In NHANES year cycle 2007–2014, sleep duration was collected from a question about the participants' routinely sleep hours: “How much sleep do you get (hours)?” In year cycle 2015–2018, sleep duration was collected from a question: “How much sleep do you usually get at night on weekdays or workdays?” Referring to previous literature (Ikonte et al., 2019; Su et al., 2021), short sleep duration was defined as ≤ 7 h per night.

The exposure variable, recreational physical activity (RPA), was collected during the household interviews utilizing the Physical Activity Questionnaire. Different from work-related physical activities (which was described as paid or unpaid work, household chores, and yard work), RPA was defined as leisure time physical engagement including sports, fitness, and other recreational activities.

Before NHANES 2007, participants reported their weekly time spent exercising, which was multiplied by the metabolic equivalent of task (MET) for that activity as defined. Since the NHANES physical activity questionnaire was changed after 2007, we chose moderate and vigorous recreational activity (MVRA) to calculate the MET-minutes per week. For detailed calculation, the participant reported minutes he or she spent on RPA in a typical day. Then, the amount of time per week for RPA was calculated by multiplying the reported number of days by the typical amount of time per day. The MVRA approach used the weighting procedure recommended in the Physical Activity Guidelines for Americans (PAGA), in which 1 min of vigorous recreational activity (VPA) was equivalent to 2 min of moderate recreational activity (MPA) (Ainsworth et al., 2000; You et al., 2022a). The time of VPA was doubled and added to the time of MPA to compute the total time.

Subsequently, we calculated the MET-minutes per week by multiplying the standard MET value of each activity by the total number of minutes per week of RPA. This physical activity quantification strategy was also used in several previous reports (Shen et al., 2019; Wilson, 2020). Each leisure activity corresponded a predetermined MET score, depending on whether reported as moderate or vigorous intensity. For instance, moderate-intensity leisure activity was regarded as 4 METs, and vigorous-intensity leisure activity was regarded as 8 METs (Ainsworth et al., 2011). Recommended by United States Department of Health and Human Services (U.S. Department of Health Human Services, 2022), participants were categorized into the following two activity levels according to their weekly RPA: insufficient to reach the guidelines (<500 MET-minutes/week) and sufficient to reach the guidelines (≥ 500 MET-minutes/week).

#### 2.2.3. Covariate assessment

Referring to previous literature (Huang et al., 2021; You et al., 2022b), demographic characteristics were extracted from the demographic questionnaire, including age, gender, race/ethnicity (Non-Hispanic white, non-Hispanic black, Mexican American, and other races), marital status (never married, married or living with partner, and widowed, divorced, or separated), family poverty income ratio [low income (<1), middle income (1,3), and high income (≥3)], and education level (below high school, high school, and college or above). In addition, the information regarding smoking status and alcohol intake status was obtained from the questionnaires of smoking cigarette use and alcohol use. According to the responses to the questionnaire, smoking status was classified as never, former, and current smoking; alcohol intake status was classified as non-drinker, moderate alcohol use, and high alcohol use. Detailed covariate information can be found at http://www.cdc.gov/nchs/nhanes/.

Moreover, we assessed the disease histories of participants in this study. Participants with systolic blood pressure (SBP) ≥140 mmHg or diastolic blood pressure (DBP) ≥90 mmHg were diagnosed as hypertension. Additionally, individuals who took drugs for hypertension or gave a positive response to the question: “Have you ever been told by a doctor or health professional that you had hypertension?” were also identified as hypertension patients. When it comes to diabetes mellitus (DM), the diagnostic criteria are as follows: (1) doctor told you have diabetes; (2) glycohemoglobin HbA1c (%) > 6.5; (3) fasting glucose (mmol/l) ≥7.0; (4) random blood glucose (mmol/l) ≥ 11.1; (5) 2-h OGTT blood glucose (mmol/l) ≥ 11.1; and (6) use of diabetes medication or insulin. The cardiovascular disease (CVD) was defined as self-reported congestive heart failure, coronary heart disease, angina, heart attack, or stroke.

### 2.3. Statistical analysis

According to the NHANES protocol, all of the data were integrated into a single dataset, and data analysis took into account the masked variance and applied the suggested weighting methodology. Sample weights from the Mobile Examination Center (MEC) interviews were re-weighted to merge 12 years' worth of survey data from NHANES 2007 to 2018 which address non-response, non-coverage, and unequal probabilities of selection; this strategy was consistent with the weight method of prior researches (Shen et al., 2019; Wilson, 2020). New weights were calculated as WT_07−18_ = (1/6) × WTMEC2YR_07−08_ + (1/6) × WTMEC2YR_09−10_ + (1/6) × WTMEC2YR_11−12_ + (1/6) × WTMEC2YR_13−14_ + (1/6) × WTMEC2YR_15−16_ + (1/6) × WTMEC2YR_17−18_, where WTMEC2YRs are variables from NHANES 2007–2018.

Participants were divided into two groups as non-depression and depression groups. In order to explore the differences between these two groups, the weighted χ2 test was utilized for categorical variables expressed as percentages, whereas the weighted linear regression model was applied for continuous variables expressed as the mean ± standard error (SE). Odds ratios (ORs) and 95% CIs were calculated for depression with RPA status in short sleep population using weighted logistic regression models. We used both unadjusted and multivariate adjusted models in this research: Crude model was adjusted for no covariates; Model 1 was adjusted for age, sex, race/ethnicity; Model 2 was adjusted for age, sex, race/ethnicity, body mass index, education marital status, poverty status, smoking status, alcohol drinking status, and disease histories.

The non-linear link between RPA and depression was evaluated using the restricted cubic spline. We further developed a two-piecewise linear regression model in order to explore the threshold effect and adjust for potential confounders. The threshold level of RPA (MET-minutes/week) was determined using a recurrence method, including identifying the inflection point along a predefined interval and choosing the inflection point that yielded the maximum likelihood model. The log-likelihood ratio test was used to compare the two-piecewise linear regression model with the one-line linear model. Simultaneously, a stratified analysis was conducted according to the impact of gender on the relationship between RPA and depression. All statistical analyses were performed with the packages of R software (http://www.R-project.org, The R Foundation). A *P*-value of < 0.05 was considered to be statistically significant.

## 3. Results

Table 1 demonstrates the characteristics of participants by depression symptoms. A total of 6,846 participants were finally included in the analysis from 58,876 participants of the 2007–2018 NHANES, and weighted participants were 52,501,159. The weighted prevalence of depression was higher in females, which took up 65.85% of all depression patients, and there was a statistically significant difference between sex (*p* < 0.001). Additionally, the weighted prevalence of depression was significantly different stratified by age, body mass index, race, marital status, family poverty income ratio, education, and smoking status (p < 0.05). The weighted prevalence of disease histories including hypertension, diabetes mellitus, and cardiovascular diseases in study participants is reported in Supplementary Table S1.

**Table 1:** Weighted characteristics of study populations in the NHANES (2007–2018) by depression status.

**Variable**	**All participants**	**Non-depression**	Depression^*^	***P-value***
Age (years)	44.22 ± 0.31	44.35± 0.31	41.72± 0.92	0.006
BMI (kg/m^2^)	28.38 ± 0.11	28.26 ± 0.11	30.60 ± 0.57	< 0.001
Sex				< 0.001
Male	54.57 (0.71)	55.62 (0.72)	34.15 (3.11)	
Female	45.43 (0.71)	44.38 (0.72)	65.85 (3.11)	
Race/ethnicity				0.002
Non-hispanic white	69.68 (1.33)	70.03 (1.34)	62.83 (2.95)	
Non-hispanic black	10.61 (0.68)	10.38 (0.68)	15.15 (1.74)	
Mexican American	6.43 (0.65)	6.34 (0.65)	8.13 (1.32)	
Other race/ethnicity	13.28 (0.64)	13.24 (0.66)	13.89 (1.58)	
Marital status				< 0.001
Never married	20.42 (0.94)	20.02 (0.97)	28.20 (2.90)	
Married/living with partner	63.60 (0.98)	64.65 (0.98)	43.19 (3.23)	
Widowed/divorced	15.98 (0.66)	15.33 (0.67)	28.62 (2.71)	
PIR				< 0.001
< 1	10.22 (0.66)	9.53 (0.66)	23.69 (2.50)	
(1,3)	30.99 (1.09)	30.42 (1.09)	41.89 (3.32)	
≥3	58.79 (1.30)	60.05 (1.28)	34.42 (3.47)	
Education				< 0.001
Below high school	2.18 (0.19)	2.08 (0.19)	4.12 (0.89)	
High school	24.65 (0.96)	24.07 (0.97)	35.80 (3.40)	
College or above	73.17 (1.02)	73.85 (1.03)	60.08 (3.53)	
Smokers				< 0.001
Never smoker	58.87 (0.85)	59.47 (0.84)	47.30 (3.92)	
Former smoker	24.58 (0.76)	24.76 (0.78)	20.99 (2.94)	
Current smoker	16.56 (0.62)	15.77 (0.63)	31.71 (3.05)	
Alcohol drinkers				0.499
Non-drinker	48.55 (0.92)	48.71 (0.95)	45.52 (3.45)	
Moderate alcohol use	31.69 (0.92)	31.67 (0.97)	31.93 (3.06)	
High alcohol use	19.77 (0.73)	19.62 (0.76)	22.55 (2.51)	
RPA (MET-min/week, continuous)	1,477.89 ± 21.53	1,488.50 ± 22.66	1,273.19 ± 75.47	0.009
RPA (MET-min/week, category)				< 0.001
< 500	27.68 (0.82)	27.09 (0.84)	39.16 (2.90)	
≥500	72.32 (0.82)	72.91 (0.84)	60.84 (2.90)	
Sleep duration (h/day)	6.32 ± 0.01	6.35 ± 0.01	5.72 ± 0.07	< 0.001

^*^Depression was measured using Patient Health Questionnaire-9 (PHQ-9), and depression was identified as PHQ-9 ≥ 10. Mean ± SE for continuous variables, and % (SE) for categorical variables. NHANES, National Health and Nutrition Examination Survey; BMI, body mass index; PIR, poverty income ratio; RPA, recreational physical activity.

Table 2 shows the association between recreational physical activity (RPA) and depression using weighted logistic regression analyses. OR (95% CI) of depression represents for two level of RPA assessed by MET-minutes/week. In the non-adjusted model, the sufficient RPA groups had a decreased risk in the odds of the development of depression [OR = 0.577 (95% Cl 0.186, 1.789)]. After adjustment for age, sex, and race/ethnicity, the odds ratio was 0.607 (0.475, 0.775). After adjusting for all confounding factors, this association persisted in Model 2 with the OR (95% CI) coming to 0.678 (0.520, 0.883) (*p* = 0.005). When grouped by gender, this association persisted in females, with OR (95% CI) = 0.633 (0.436, 0.920). However, in males, this positive association was not significant, with OR (95% CI) = 0.771 (0.474, 1.254). Furthermore, we examined the interactions between RPA and gender in the fully adjusted model (Model 2), while no significant association was found (p for interaction = 0.546).

**Table 2:** Associations between recreational physical activity and depression in short sleepers.

**RPA (MET-min/week)**	**Crude model** ^**a**^		**Model 1** ^**b**^		**Model 2** ^**c**^	
	**OR (95% CI)**	***P-*value**	**OR (95% CI)**	***P-*value**	**OR (95% CI)**	***P-*value**
< 500	Reference		Reference		Reference	
≥500	0.577 (0.186, 1.789)	< 0.001	0.607 (0.475,0.775)	< 0.001	0.678 (0.520,0.883)	0.005
**Male**						
< 500	Reference		Reference		Reference	
≥500	0.768 (0.170, 3.463)	0.250	0.738 (0.476,1.145)	0.179	0.771 (0.474,1.254)	0.298
**Female**						
< 500	Reference		Reference		Reference	
≥500	0.563 (0.187, 1.697)	0.002	0.549 (0.389,0.775)	0.001	0.633 (0.436,0.920)	0.019

a Crude model, no covariates were adjusted.

b Model 1: age, sex, and race/ethnicity were adjusted.

c Model 2: age, sex, race/ethnicity, body mass index, education, marital status, poverty status, smoking status, alcohol drinking status, and disease histories were adjusted.

OR, odds ratio; CI, confidence interval.

**Figure 1:**
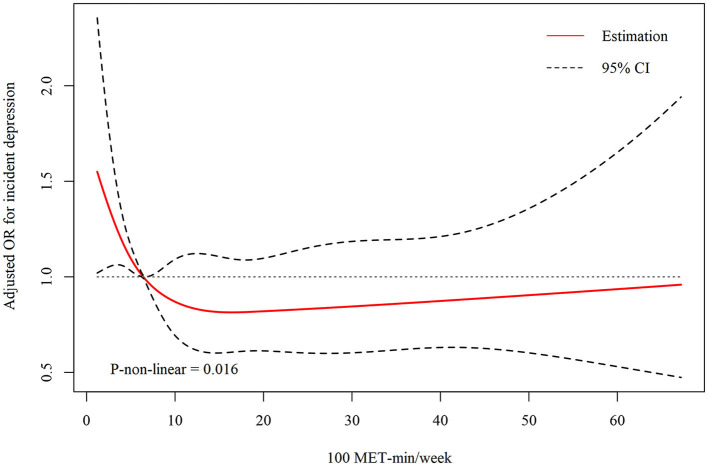
Dose–response relationship between recreational physical activity and depression in short sleepers.

Since the amount of RPA was a continuous variable, the analysis of non-linear relationship was necessary. To better describe the relationship, the unit of RPA was represented as 100^*^ MET-minutes/week in the Figure 1. In the present study, we found that the relationship between RPA and depression was non-linear (after adjusting age, sex, race/ethnicity, body mass index, education marital status, poverty status, smoking status, alcohol drinking status, and disease histories, the *p*-value for non-linearity was 0.016). In this figure, the red solid line indicated the OR of depression, and the dotted lines represented the point-wise 95% confidence interval.

Log-likelihood ratio test was conducted to compare the one-line (non-segmented) model to segmented regression model, and our results showed that threshold was existed. By two-piecewise linear regression model, we calculated the inflection point was 640 MET-minutes/week. On the left of inflection point, the OR, 95%CI and *P*-value were 0.891 (0.834, 0.953) and < 0.001, respectively. However, we observed no relationship between RPA and depression score on the right of inflection point, with OR, 95%CI and *P*-value as 0.999 (0.990, 1.009) and 0.891 (Table 3).

**Table 3:** Threshold effect analysis of relationship between recreational physical activity and depression in short sleepers.

**Outcome**	**OR (95% CI)**	***P-*value**
One—line linear regression model	0.991 (0.983, 1.000)	0.043
**Two—piecewise linear regression model**		
RPA < 640 (MET-min/week)	0.891 (0.834, 0.953)	< 0.001
RPA ≥ 640 (MET-min/week)	0.999 (0.990, 1.009)	0.891
Log—likelihood ratio test		0.002

Adjusted for age, sex, race/ethnicity, body mass index, education, marital status, poverty status, smoking status, alcohol drinking status, and disease histories.

Furthermore, we examined this relationship in different male and female groups. In male groups (Supplementary Table S2), the threshold effect also applied with p-value of log-likelihood ratio test as 0.032, and the inflection point was 480 MET-minutes/week, which was a bit lower than the point showed in the total sample. For female groups (Supplementary Table S3), the *p*-value of log-likelihood ratio test was 0.014, which indicated that threshold effect also persisted in this population and the inflection point was 780 MET-minutes/week. Two piecewise linear regression model showed a consist negative trend in the relationship between RPA and depression in the female group.

## 4. Discussion

Elucidating the relationship between recreational physical activity (RPA) and depression status in short sleep population may inform our understanding of the clinical relevance of specific changes in lifestyle behavior, especially the RPA situation. As far as we know, ours was the first to explore the associations and dose–response relationship between RPA and depressive symptoms in the specific short sleep populations. In our analysis, the risk of depressive symptoms was negatively associated with physical activity status. In addition, the stratified analyses showed that this association was more significant in females. Furthermore, a non-linear dose–response relationship was found between RPA and depressive symptoms.

Plentiful evidence has demonstrated that short sleep duration might lead to increased risk of depression (Sun et al., 2018; Ogawa et al., 2019); however, it was not well-characterized whether RPA would still be effective in this situation. Our results found that there was a significant association between RPA and depression in all three models, which indicated that maintaining a certain amount of RPA was a protective factor to depression, even with short sleep status. However, there was a saturation effect of RPA to depression in short sleep population. From the threshold analysis, it seemed that doing no or excessive RPA was associated with risk of depression, and before the RPA amount of 640 MET-minutes/week, increasing exercise or sports activities might significantly help to decrease this risk. Considering that the MET for moderate-intensity activity (e.g., brisk walking, slow cycling, or tennis doubles) was 4 (Jeong et al., 2019), one way to reach 640 MET-minutes per week was to stick to 160 min brisk walk in a week.

The underlying molecular mechanisms concerning to the association between short sleep and depression may be concluded to the activation of hypothalamus pituitary adrenal (HPA) system (Rao et al., 2009), the functional change of 5-hydroxytryptamine (5-HT) system (Tsao et al., 2006), and the increase in inflammatory cytokine production (Dolsen et al., 2020). Of note, the role of recreational activity such as physical exercise in preventing or improving depressive symptoms was also related to these factors, and specific exercise types may have different effects (You et al., 2022). Exercise as a treating intervention in depression patients was mainly based on the theory of kinematics, neuron effect, and sports psychology (You et al., 2021a). The stress reaction of depression was related to the HPA axis induced by brain, and animal studies showed that physical exercise can reduce the increase in corticosterone (CORT) and the decrease in glucocorticoid receptor (GR) (Zheng et al., 2006). Serotonin signaling pathway was another exercise factor that can lead to the improvement in depressive symptoms. The regulation in blood serotonin after exercise was similar to the effects of selective serotonin reuptake inhibitors (Wipfli et al., 2011; Yuan et al., 2015). In addition, evidence also proved that biomarkers of inflammation were also potential mediators of the relationship between exercise and depressive symptoms (Booij et al., 2015; Zepf et al., 2016).

By using the threshold analysis, our study also found that gender had impact on the relationship between RPA and depression in the short sleep population. Although accumulated findings have not been entirely consistent, much evidence proved that sociodemographic factors including race, education, and marriage could affect depression in both men and women (Zimmerman and Katon, 2005; Van de Velde et al., 2010). From the perspective of social status and roles, men were more engaged in physical labor, and more work activity might make it difficult for them to participate in more recreational activities under the condition of lack of sleep. In this condition, for the male population engaged in high physical labor, we affirmed the positive significance of appropriate volume of recreational activities, but the premise was to ensure sufficient sleep hour rather than excess recreational activities, which would be the primary strategy to reduce depressive symptoms in this group. Compared to males, several females not only had to deal with full-time paid jobs but also did most of the child care and domestic work at home. In modern society, women were increasingly “sandwiched” between caring for young children and caring for sick and elderly family members, which meant that lacking of sleeping and recreational time might lead to higher prevalence of depression in this group. In this situation, appropriate volume of exercise or recreational activities may help women to cope with deficiency of physical activity and release pressure.

There was no formula as to what type and intensity of RPA performed best to reduce depression risks for the short sleep population, but aerobic exercise approximately three times per week has been found to treat symptoms (Stanton and Reaburn, 2014). Aerobic exercise has been proved to be efficient to improve sleep quality (Reid et al., 2010; Passos et al., 2011) and reduce risk factors for short sleep (Stockelman et al., 2021). Evidence showed that for depression groups, aerobic exercise might be considered as a promising strategy for improving anxiety and depressive symptoms (Bailey et al., 2018; Wegner et al., 2020). In addition, accumulating evidence also suggested that team sports were effective in diminishing risk and promoting rehabilitation for depressive symptoms (Boone and Leadbeater, 2006; Sabiston et al., 2013). Recently, there was a debate on whether high-intensity activity like high-intensity interval exercise (HIIE) was an appropriate strategy in countering depression (You et al., 2021b). However, excessive intensity exercise may cause more fatigue for short sleepers, and in consist with our analysis, HIIT program was too arduous and could evoke experiences of incompetence, failure, and lower self-esteem, thus reducing anti-depression effects (Robertson-Wilson et al., 2017). In short, moderate aerobic activities involving group participation could be recommended as feasible options for countering depression in short sleep population, while the detailed intervention strategy should be verified by further high-quality randomized controlled trials (RCTs).

The strengths of our study were that the data we analyzed were from a relatively large nationally representative sample of US adults, and the dose–response relationship and the stratified analyses by gender were conducted to explore the association between RPA and depression in short sleep groups. However, there were also some limitations. First, due to the cross-sectional nature, it was unable for us to infer causality from the results. Although this article represented one of the first attempts to investigate the role of RPA with the associations of depression in short sleep population, further studies might consider the investigation of this issue among various subpopulations (such as teenagers or minorities) and better explore which type and intensity of RPA could prevent or improve one's depressive symptoms. Second, the self-report measurement of RPA and sleep hour was another concern due to the potential reporting bias. Finally, there might be some unconsidered potential factors that affected the study results, despite large samples from a national representative survey. In future, additional cohort studies should further consider the nuanced factors and verify the results of this study.

## 5. Conclusion

To sum up, RPA volume was independently associated with the incidence of depression in the short sleep population based on the National Health and Nutrition Examination Survey from 2007 to 2018. A dose–effect relationship was found, which revealed a U-shaped relationship between RPA bouts and depression. For general short sleepers, maintaining the RPA volume approximately 640 MET-minutes/week (equal to 80-min vigorous physical activity or 160-min moderate physical activity per week) was conducive to the lower depression risks. Gender difference should be considered as an important factor for further studies to examine these relationships and explore mechanisms. Achieving a balance between exercise and sleep can help promote physical and mental health and prevent depression.

## Data availability statement

Publicly available datasets were analyzed in this study. This data can be found here: https://www.cdc.gov/nchs/nhanes/.

## Ethics statement

The studies involving human participants were reviewed and approved by National Center for Health Statistics Research Ethics Review Board. The patients/participants provided their written informed consent to participate in this study.

## Author contributions

YY, MW, YC, and XM: conceptualization. YY and MW: methodology. YY and YC: software and formal analysis. YF, JL, and AA: investigation. YY, MW, and YC: writing—original draft preparation. YY, MW, YC, YF, and AA: writing—reviewing and editing. JL and XM: supervision. All authors have read and agreed to the published version of the manuscript.
