# Supplementary material for: The association between recreational physical activity and depression in the short sleep population: a cross-sectional study
Source: Front Neurosci. 2023 May 25;17:1016619. doi: 10.3389/fnins.2023.1016619 (PMC10248511; doi:10.3389/fnins.2023.1016619)
Supplement: Supplementary file 1 [file Table_1.docx]

Table S1. Weighted characteristics and disease histories of study populations in the NHANES (2007 - 2018) by depression status.

|  | All participants | | Non-depression | | Depression | P-value |
| --- | --- | --- | --- | --- | --- | --- |
| Hypertension |  | | | | | 0.002 |
| No | 68.04 (0.76) | 68.51 (0.77) | | 58.98 (3.17) | |  |
| Yes | 31.96 (0.76) | 31.49 (0.77) | | 41.02 (3.17) | |  |
| DM |  | | | | | 0.067 |
| No | 90.16 (0.47) | 90.34 (0.51) | | 86.80 (1.91) | |  |
| Yes | 9.84 (0.47) | 9.66 (0.51) | | 13.20 (1.91) | |  |
| CVD |  | | | | | 0.002 |
| No | 94.53 (0.38) | 94.74 (0.39) | | 90.48 (1.65) | |  |
| Yes | 5.47 (0.38) | 5.26 (0.39) | | 9.52 (1.65) | |  |

Notes: DM, diabetes mellitus; CVD, cardiovascular diseases.
