# Supplementary material for: The association between recreational physical activity and depression in the short sleep population: a cross-sectional study
Source: Front Neurosci. 2023 May 25;17:1016619. doi: 10.3389/fnins.2023.1016619 (PMC10248511; doi:10.3389/fnins.2023.1016619)
Supplement: Supplementary file 2 [file Table_2.docx]

Table S2. Threshold effect analysis of relationship between recreational physical activity and depression in male group of short sleepers.

| Outcome | OR (95% CI) | *P-value* |
| --- | --- | --- |
| One - line linear regression model | 0.991 (0.979, 1.003) | 0.159 |
| Two - piecewise linear regression model |  |  |
| RPA < 480 (MET-minutes/week) | 0.816 (0.687, 0.969) | 0.021 |
| RPA ≥ 480 (MET-minutes/week) | 0.997 (0.984, 1.010) | 0.638 |
| Log - likelihood ratio test |  | 0.032 |

Notes: adjusted for age, race/ethnicity, body mass index, education, marital status, poverty status, smoking status, alcohol drinking status and disease histories.
