# Supplementary material for: The association between recreational physical activity and depression in the short sleep population: a cross-sectional study
Source: Front Neurosci. 2023 May 25;17:1016619. doi: 10.3389/fnins.2023.1016619 (PMC10248511; doi:10.3389/fnins.2023.1016619)
Supplement: Supplementary file 3 [file Table_3.docx]

Table S3. Threshold effect analysis of relationship between recreational physical activity and depression in female group of short sleepers.

| Outcome | OR (95% CI) | *P-value* |
| --- | --- | --- |
| One - line linear regression model | 0.990 (0.978, 1.002) | 0.109 |
| Two - piecewise linear regression model |  |  |
| RPA < 780 (MET-minutes/week) | 0.915 (0.858, 0.975) | 0.006 |
| RPA ≥ 780 (MET-minutes/week) | 1.095 (1.019, 1.176) | 0.013 |
| Log - likelihood ratio test |  | 0.014 |

Notes: adjusted for age, race/ethnicity, body mass index, education marital, status, poverty status, smoking status, alcohol drinking status and disease histories.
