# Supplementary figures and images for: The association between recreational physical activity and depression in the short sleep population: a cross-sectional study
Source: Front Neurosci. 2023 May 25;17:1016619. doi: 10.3389/fnins.2023.1016619 (PMC10248511; doi:10.3389/fnins.2023.1016619)

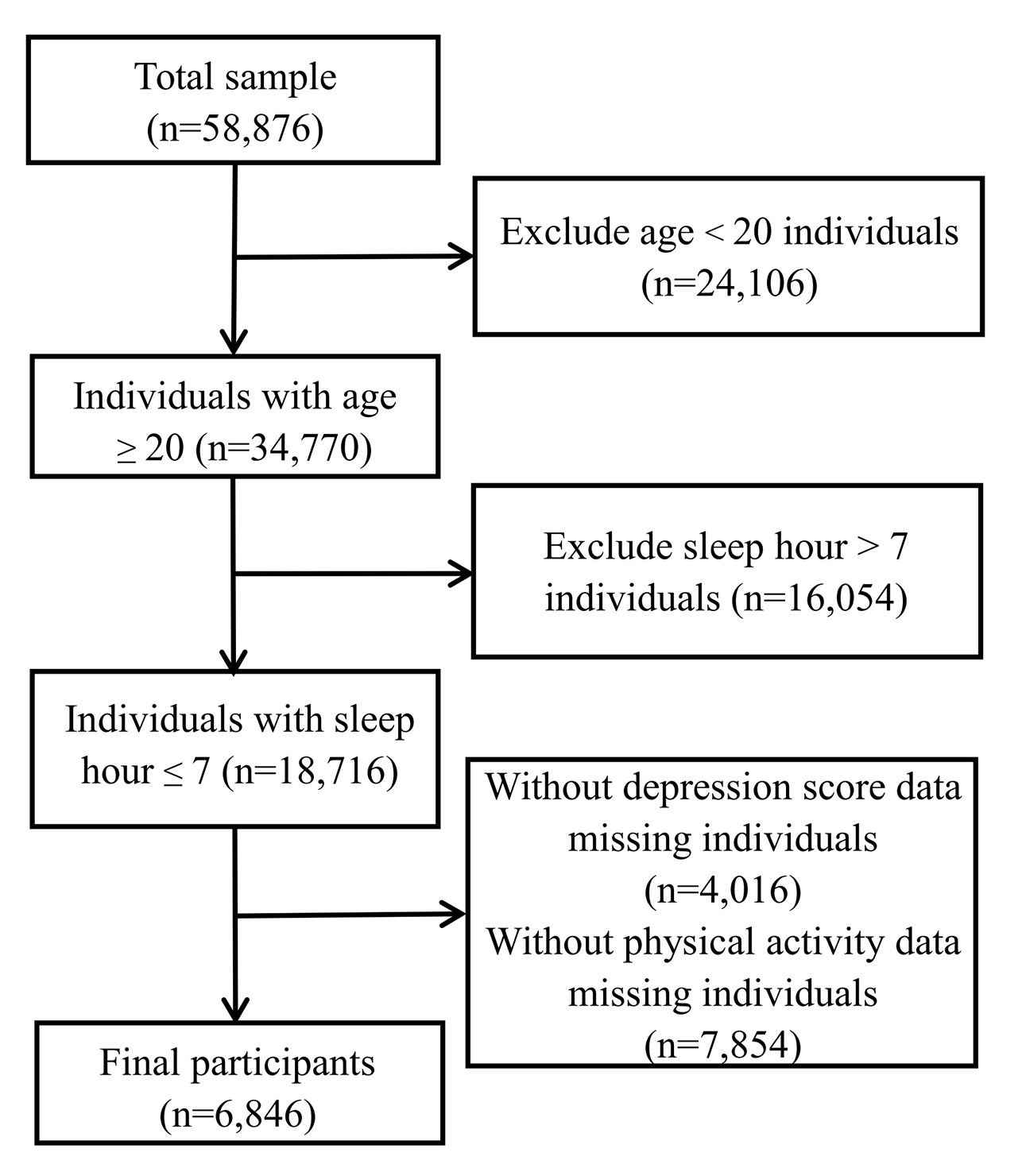

Supplement: Supplementary file 4 [file Image_1.TIF]
